# A Novel Ship-Tracking Method for GF-4 Satellite Sequential Images

**DOI:** 10.3390/s18072007

**Published:** 2018-06-22

**Authors:** Libo Yao, Yong Liu, You He

**Affiliations:** 1 Research Institute of Information Fusion, Naval Aviation University, Yantai 264001, China; ylb_rs@126.com; 2 School of Electronic Science, National University of Defense Technology, Changsha 410073, China; xhliuyong@sina.com

**Keywords:** GF-4 satellite, ship detection and tracking, RPCs correction, data association

## Abstract

The geostationary remote sensing satellite has the capability of wide scanning, persistent observation and operational response, and has tremendous potential for maritime target surveillance. The GF-4 satellite is the first geostationary orbit (GEO) optical remote sensing satellite with medium resolution in China. In this paper, a novel ship-tracking method in GF-4 satellite sequential imagery is proposed. The algorithm has three stages. First, a local visual saliency map based on local peak signal-to-noise ratio (PSNR) is used to detect ships in a single frame of GF-4 satellite sequential images. Second, the accuracy positioning of each potential target is realized by a dynamic correction using the rational polynomial coefficients (RPCs) and automatic identification system (AIS) data of ships. Finally, an improved multiple hypotheses tracking (MHT) algorithm with amplitude information is used to track ships by further removing the false targets, and to estimate ships’ motion parameters. The algorithm has been tested using GF-4 sequential images and AIS data. The results of the experiment demonstrate that the algorithm achieves good tracking performance in GF-4 satellite sequential images and estimates the motion information of ships accurately.

## 1. Introduction

**Figure 1:**
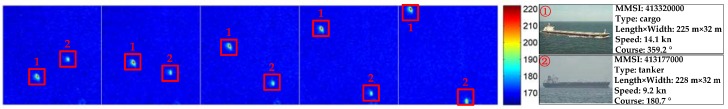
Two ships in five sequential images from the GF-4 satellite and detailed AIS information of ships.

The geostationary orbit (GEO) remote sensing satellite has become a very important aspect of current remote sensing areas because of its advantage of wide-swath scanning, near real-time continuous observation and rapid operational response, and has tremendous potential in maritime target surveillance, which needs simultaneously wide coverage and high temporal resolution. The Chinese GF-4 satellite, launched in 2015, is the first array staring-imaging optical remote sensing satellite and has the highest spatial resolution in GEO remote sensing satellites at present. The staring camera on the GF-4 satellite has the highest spatial resolution (50 m ground sample distance, GSD) in visible and near infrared (VNIR) band, the widest imaging coverage (400 km × 400 km) and the highest temporal resolution (20 s) of any similar existing systems [1]. The spatial resolution of 50 m is much lower than the high resolution (≤5 m) of remote sensing satellites in low Earth orbit (LEO), but it is good enough to track large ships in near-real time from space. The staring camera, almost like a video camera, has high responsiveness and high-revisit observation capabilities. It cannot only adjust to the observation area within a few minutes, but also features high-frequency continuous imaging of the same area [2]. These new features enable the GF-4 satellite to play a significant role in many applications, especially in marine surveillance, which can be used for ship traffic surveillance, coastal management, and military purposes [3]. Therefore, this paper presents a complete processing algorithm for ship tracking using GF-4 satellite sequential images. The consecutive image sequences of GF-4 satellites can be used to obtain ships’ motion information, which can assist decision-making, guide high-resolution LEO satellites for further recognition and identification, and so on. The GEO remote sensing satellite has become a new powerful tool in maritime surveillance. Recently, automatic identification system (AIS) satellite constellations have been available for cooperative ships’ surveillance. However, in piracy, terrorism, illegal weapons proliferation, irregular immigration, drug smuggling, illegal fisheries, and other activities, the AIS equipment of non-cooperative ships are intentionally turned off or camouflaged for deception. Besides, military ships always deliberately try to hide. Therefore, the GEO remote sensing satellite can help find ships which pose a threat, or are known to engage in illegal activities, especially for continuous observing. So far, target-tracking methods using image sequences are mostly designed for ground and aerial videos, which have narrow field of view (FOV), long observation time and the targets have richer features (e.g., shape, texture) with a high spatial resolution. While satellite images have wider FOV, lower spatial resolution and more targets in one scene compared to ground and aerial videos. Targets in satellite images have fewer pixels, less detail and lower discrimination. Targets motion parameters estimation using LEO remote sensing satellite images has been studied in recent years. Most high resolution optical remote sensing satellites carry panchromatic (PAN) and multispectral (MS) sensors that are in different positions. Then there is a slight time lag between the acquisitions of the PAN and MS images, which can be used to extract the moving information of targets. The single-pass imagery has been successfully used to extract motion information of vehicles and planes [4,5,6,7,8,9,10,11], while the time lag is too small to detect low speed moving vessels. Motion parameters can also be estimated using along-track sequential or stereo satellite images, which are taken by agile remote sensing satellites such as WorldView-2 with multi-angular look capability [12,13,14,15]. More recently, Skysat, Jilin-1 and Zhuhai-1 small satellite constellations, which can record short videos, have been successfully tested for tracking moving targets [16,17,18,19]. However, limited to the LEO satellite’s high pass speed and long revisit period, moving targets can only be observed in a short time interval and only a few plots or a short track can be obtained. In addition, most LEO remote sensing satellites have a narrow swath which is usually tens of kilometers. The limitations of LEO remote sensing satellites restrict their use for maritime surveillance to some extent. The GEO remote sensing satellite can provide medium spatial resolution, wide scan coverage and short revisit interval image series. Target-tracking methods for GEO remote sensing satellites have been discussed in some published literatures [3,20,21,22]. However, compared to ground, aerial and satellite videos, the GEO remote sensing satellite sequential images have much lower spatial resolution. As shown in Figure 1, two ships are moving in five sequentially near infrared (NIR) band images from the GF-4 satellite (the size of chips is 128 pixels × 128 pixels and acquisition Time (UTC) is given in Table 1), and some automatic identification system (AIS) information of two ships is shown in the right part of this figure. We can see that the ships have fewer pixels and lower discrimination, and ships can be regarded as weak point targets in the sea background.

Meanwhile, the imaging interval of the GF-4 satellite (from tens of seconds to a few minutes per frame) is long, compared to traditional videos, and satellite images are also affected by light and other factors, resulting in higher false rate of detection. These new characteristics and problems of the GF-4 satellite cannot be directly solved with traditional video target-tracking methods. Image sequences of the GF-4 satellite are more suitable for direct detection processing for each frame, and then multi-frame multi-target data association processing. The accuracy positioning of ships using the GF-4 satellite is also important for ship tracking. Due to the long distance away from Earth, a small attitude control error of the GEO platform will cause a large position bias between the target corresponding to pixel point and image position. By directly using rational polynomial coefficients (RPCs) for geometric calibration, the absolute positioning accuracy of the GF-4 satellite’s 1A level image is about 4 km. The large positioning error will affect ships association and tracking accuracy. At the same time, the motion of the space-borne camera will make the position between frames deviate. These problems can be resolved by means of ground control points (GCPs). However, the surveillance area is at sea and there are few or even no GCPs in sea area. AIS is an identification system that transmits a ship’s identification, position and other critical information that can be used to assist in navigation, improve maritime safety and track ships for maritime domain awareness, search and rescue, environmental monitoring, and maritime intelligence application. Thanks to the wide use of AIS in marine surveillance, this paper uses AIS information as dynamic GCPs to register each frame to obtain accurate target position information without inter-frame registration.

A method of ship detection and tracking for GEO remote sensing satellite sequential images is designed in this paper. The rest of this paper is organized as follows: the processing framework is described in Section 2. Experiments and results using GF-4 satellite sequential images are presented in Section 3. Finally, discussion and conclusion are presented in Section 4.

## 2. Proposed Method Framework

**Figure 2:**
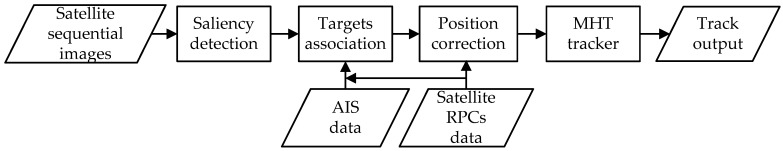
Proposed method framework.

The flowchart of the proposed ship tracking algorithm is illustrated in Figure 2. The algorithm consists of three steps, which are ship detection, position correction and ship tracking. First, ship detection is realized by a local visual saliency map based on peak signal: noise ratio. In addition, then the ship position is corrected by the RPCs model using AIS data as GCPs. To correct the error of RPCs, AIS data are correlated with the detection of GF-4 satellite in the image coordinates, and then the associated point pairs are used to compensate the error parameters. Finally, an improved MHT framework with amplitude assisted is applied to track ships and estimate the ships’ motion parameters. The algorithm is introduced below in detail.

### 2.1. Ship Detection

As the reflectance of water is weakest in the NIR band of the GF-4 satellite image, the contrast between a ship and its surrounding water is strongest among five bands. Therefore, the NIR band is selected for ship detection.

The spatial resolution of the GF-4 satellite is much lower than that of LEO remote sensing satellites, so ships can be regarded as low observable targets in the GF-4 satellite images. As the intensity distribution of sea background clutter is not uniform in different regions in the same frame, it is not good to use the global threshold to detect targets directly. Therefore, this paper proposes a local saliency map algorithm for detection based on PSNR. The PSNR quantifies the intensity at the peak of the target’s point relative to the noise in the background, so the local saliency value of a pixel (x,y) in an image is defined as

(1) $$sal(x,y)=\frac{I(x,y)-\mu_{b}}{\sigma_{b}}$$

where I(x,y) is the amplitude at (x,y), $\mu_{b}$ and $\sigma_{b}$ are the mean and standard variance of pixel intensities in the background around (x,y), respectively. To better calculate the statistical properties of the background, two rectangular windows (an inner window and an outer window) centralized by (x,y) are used, and the statistical characteristics of pixels between two windows are calculated. Considering the resolution of the GF-4 satellite image and the size of the ship, the size of the outer window is set to 20 pixels, and the size of the inner window is set to 10 pixels, empirically. To get the binary image D of detection results, a threshold th is used. If the saliency value of a pixel is not less than th, it is determined as a potential target, thus the binary image is gotten as

(2) $$D(x,y)=\left\{ \begin{array}{l} 1,sal(x,y)\geq th\\ 0,\mathrm{else} \end{array}\right.$$

After the threshold segmentation, we use connected component labeling to separate the blobs and filter out ones, which are not likely to be ship candidates based on the size criteria. In this paper, only the blobs whose pixel sizes are between 2 and 50 are kept. A blob’s centroid is calculated as the location, and the maximum amplitude of a blob is calculated as the amplitude of the target.

### 2.2. Position Correction

#### 2.2.1. RPCs Model

RPCs model is the most popular method for geometry calibration of satellite images. It is a simple and generalized model that can obtain approximately the same accuracy as the strict geometric imaging model. The image coordinate is represented as ratios of the cubic polynomials in the ground coordinates and its form is as follows:

(3) $$\begin{array}{l} y=\frac{{\mathrm{Num}}_{L}(u,v,w)}{{\mathrm{Den}}_{L}(u,v,w)}\\ x=\frac{{\mathrm{Num}}_{S}(u,v,w)}{{\mathrm{Den}}_{S}(u,v,w)} \end{array}$$

with

(4) $$u=(\varphi -\varphi_{0})/\varphi_{s},v=(\lambda -\lambda_{0})/\lambda_{s},w=(h-h_{0})/h_{s},y=(l-l_{0})/l_{s},x=(s-s_{0})/s_{s}$$

where (u,v,w) and (x,y) are the normalized ground and image coordinates respectively. (φ,λ,h) is geodetic latitude, longitude, and height, (l,s) are the line and sample in image coordinates. $\varphi_{0},\lambda_{0},h_{0},l_{0},s_{0}$ and $\varphi_{s},\lambda_{s},h_{s},l_{s},s_{s}$ are the offset and scale factors for latitude, longitude, height, line, and sample, separately. The Num and Den are rational functions whose form as follows:

(5) $$\begin{array}{l} p(u,v,w)=a_{1}+a_{2}v+a_{3}u+a_{4}w+a_{5}vu+a_{6}vw+a_{7}uw+a_{8}v^{2}+a_{9}u^{2}+a_{10}w^{2}+\\ a_{11}uvw+a_{12}v^{3}+a_{13}vu^{2}+a_{14}vw^{2}+a_{15}v^{2}u+a_{16}u^{3}+a_{17}uw^{2}+a_{18}v^{2}w+a_{19}u^{2}w+a_{20}w^{3} \end{array}$$

where $a_{1}\sim a_{20}$ are the coefficients of RPCs model. As ships are at sea, the elevation h approximates to 0. The corresponding ground coordinates can be calculated using the inverse transformation of Equation (3) which can be solved by Taylor series expansions using a certain number of iterative solutions.

After ship detection, the image coordinates of ships can be obtained, and the ground coordinates of ships can be estimated using the RPCs model. However, the initial ground coordinates are inaccurate for direct geo-referencing of images because of the systematic error of RPCs. To correct the system error, the image error compensation model is introduced. The commonly used bias-compensated RPCs model is an affine transformation function, which is expressed as

(6) $$\begin{array}{l} l^{\prime }=e_{0}+e_{1}l+e_{2}s\\ s^{\prime }=f_{0}+f_{1}l+f_{2}s \end{array}$$

where $\left(l^{\prime },s^{\prime }\right)$ and (l,s) are image coordinates obtained from RPCs and tie points respectively, $\left(e_{i},f_{i}),(i=0,1,2\right)$ are the transformation parameters. The key to get transformation parameters is to get the point pairs. In this paper, we use the AIS data which are associated with GF-4 detections as the control points.

#### 2.2.2. Ship Association

Before ship association between AIS data and GF-4 detections, a time projection step for AIS data should be implemented as AIS transmissions occur continuously. In the paper, the positions of AIS data are linearly interpolated or extrapolated to derive ship positions at GF-4 satellite image acquisition time. As there is an approximate 40 s time lag between the acquisition time of the image in NIR band and the acquisition time given in the metadata file [3], the delay should be added in the time projection of AIS data. A position $y_{i}^{}$ of AIS data in the image coordinates can be obtained by inverse transformation of Equation (3) and the provided RPCs. As global nearest neighbor (GNN) is one of the most prevailing algorithms for data association, we use it for ship association. GNN assignment seeks the best association with the lowest global cost and allows each point to be assigned to only one track. During the GNN association process, the statistical distance $d_{ij}$ from a AIS position $y_{i}^{}$ to a GF-4 detection point $x_{j}^{}$ as cost function would be calculated. The weighted sum of distances should be minimized,

(7) $$\min\sum_{j=1}^{N_{GF4}}\sum_{i=1}^{N_{AIS}}d_{ij}T_{ij},\;d_{ij}=\left\|y_{i}^{}-x_{j}^{}\right\|$$

and the following constraints should be met:

(8) $$\left\{ \begin{array}{l} d_{ij}\leq \varepsilon ,i↔j\\ \sum_{i=1}^{N_{AIS}}T_{ij}\leq 1,\sum_{j=1}^{N_{GF4}}T_{ij}\leq 1,T_{ij}\in \{0,1\} \end{array}\right.$$

where $N_{GF4}$ and $N_{AIS}$ are the number of GF-4 detections and AIS tracks, respectively. ε is the maximum distance and $T_{ij}$ is the associated variable. If the distance between two points is less than ε, the target is said to be validated for a given point. $T_{ij}=1$ means point-to-point association while $T_{ij}=0$ means no association. The Munkres (also known as Hungarian) algorithm is an efficient algorithm to solve the assignment problem, and it is the easiest one to implement and optimality is guaranteed comparing to other optimal searching methods such as Jonker-Volgenant-Castano (JVC) and Auction algorithms. Therefore, the corresponding relationship in GNN assignment is obtained by Munkres algorithm.

As the error correction model of ship position is an affine transformation function, only 3 pairs of matching points can solve the affine parameters, and more point pairs can be used by least squares (LS). However, many point pairs obtained by ship association are incorrect. If all the input data are treated indiscriminately directly for solving the affine transformation parameters, there will be a large parameter estimation error. Therefore, a matching subset suitable for estimating the affine transformation must be automatically selected. To robustly estimate the parameters in system error, this paper uses the random sample consensus (RANSAC) algorithm to remove the erroneously associated point pairs, and robust estimation of affine parameters is achieved through LS. The steps are as follows:

(1) Each time 3 pairs of matching points are randomly selected to calculate the affine transformation parameters, and then obtain the error of other point pairs under the current transformation. The point pair whose error is smaller than a certain threshold is used as the interior point, and the interior point set is saved;
(2) Repeat random sampling to get the maximum interior point set;
(3) The LS algorithm is used to solve the affine transformation parameters for the maximum interior point set. When solving the affine transformation parameters, a point position of AIS is referred $\left(l^{\prime },s^{\prime }\right)$, and the corresponding matching point of GF-4 detection is referred (l,s).

After the offset compensation of RPCs model is achieved, the forward transform of GF-4 detection is used to realize the transformation from image coordinates to geographic coordinates in a single frame image.

### 2.3. Ship Tracking 

#### 2.3.1. Ship Modeling

To track ships using GF-4 satellite, an appropriate ship modeling is required before ship tracking. As the resolution of GF-4 satellite is far lower than that of LEO imaging satellites, ships are small and weak, and it is difficult to extract rich features. In this paper, only the target position and target amplitude are used for tracking in each frame. The measurement at time *k* can be expressed as

(9) $$z_{k}={\left(lon_{k},lat_{k},amp_{k}\right)}^{T}$$

where $lon_{k}$ and $lat_{k}$ are the longitude and latitude of the measurement in geographic coordinates, $amp_{k}$ is the maximum amplitude of the target in the image. The state of a ship is defined at time *k* as

(10) $$x_{k}={\left(\lambda_{k},{\dot{\lambda }}_{k},\varphi_{k},{\dot{\varphi }}_{k},a_{k}\right)}^{T}$$

where $\lambda_{k},\varphi_{k}$ and ${\dot{\lambda }}_{k},{\dot{\varphi }}_{k}$ represent the position and velocity components along longitude and latitude directions, respectively. The basic unit of longitude, latitude and course is the degree (°), and the velocity is determined in knots (kn). $a_{k}$ is the amplitude. The measurement equation information is expressed as

(11) $$z_{k}=H_{k}x_{k}+w_{k},w_{k}\sim N(0,R_{k})$$

where

(12) $$H_{k}=\left[\begin{array}{ccccc} 1 & 0 & 0 & 0 & 0\\ 0 & 0 & 1 & 0 & 0\\ 0 & 0 & 0 & 0 & 1 \end{array}\right]$$

(13) $$R_{k}=\mathrm{diag}(\sigma_{p}^{2},\sigma_{p}^{2},\sigma_{a}^{2})$$

where $H_{k}$ is the observation matrix, $R_{k}$ is the noise matrix, and $\sigma_{p}$ is the standard deviation of positioning error after correction of position offset both in longitude and latitude. $\sigma_{a}$ is the standard deviation of the amplitude. The speed $s_{k}$ and the course $\theta_{k}$ over ground (relative to true north) can be obtained that

(14) $$s_{k}=\sqrt{{\dot{\lambda }}_{k}{}^{2}+{\dot{\varphi }}_{k}{}^{2}}$$

(15) $$\theta_{k}=\left\{ \begin{array}{l} \arctan({\dot{\lambda }}_{k}/{\dot{\varphi }}_{k}),\;\;\;\;\;\;\;\;\;{\dot{\lambda }}_{k}\geq 0,{\dot{\varphi }}_{k}>0\;\\ 360{}^{\circ}+\arctan({\dot{\lambda }}_{k}/{\dot{\varphi }}_{k}),{\dot{\lambda }}_{k}<0,{\dot{\varphi }}_{k}>0\;\\ 180{}^{\circ}+\arctan({\dot{\lambda }}_{k}/{\dot{\varphi }}_{k}),\;\;\;\;\;\;\;\;\;\;{\dot{\varphi }}_{k}<0\;\;\\ 90{}^{\circ},\;\;\;\;\;\;\;\;\;\;\;\;\;\;\;\;\;\;\;\;\;\;\;{\dot{\lambda }}_{k}>0,{\dot{\varphi }}_{k}=0\\ 270{}^{\circ},\;\;\;\;\;\;\;\;\;\;\;\;\;\;\;\;\;\;\;\;\;\;{\dot{\lambda }}_{k}<0,{\dot{\varphi }}_{k}=0 \end{array}\right.$$

The state transition model in the dead reckoning of a rhumb line track from one point $\left(\lambda_{1},\varphi_{1}\right)$ to the other point $\left(\lambda_{2},\varphi_{2}\right)$ can be expressed as

(16) $$\left\{ \begin{array}{l} \varphi_{2}=\varphi_{1}+\dot{\varphi }T/60\\ \lambda_{2}=\lambda_{1}+\dot{\lambda }T\sec(\varphi_{1})/60 \end{array}\right.$$

where T is the time interval, 1/60 is the factor used to convert the length (nm) to degree (°) since 1° of latitude equals approximately 60 nm. The motion model can be described as

(17) $$x_{k}=f_{k-1}(x_{k-1})+G_{k-1}v_{k-1}$$

where $f_{k-1}$ is state transition function, $G_{k-1}$ and $v_{k-1}$ is the process noise matrix and process noise, respectively. We define $v_{k-1}={\left({\ddot{\lambda }}_{k-1},{\ddot{\varphi }}_{k-1},0\right)}^{T}$ and $v_{k-1}\sim N(0,B_{k})$, which represents a multivariate Gaussian function with zero mean and covariance $B_{k}$,

(18) $$B_{k}=\mathrm{diag}(\sigma_{v}^{2},\sigma_{v}^{2},0)$$

where $\sigma_{v}$ is the process noise standard deviation. Derived from Equation (17), the state transition equation is

(19) $$x_{k}=\left[\begin{array}{c} \begin{array}{c} \lambda_{k-1}+{\dot{\lambda }}_{k-1}T_{k-1}\sec(\varphi_{k-1})/60\\ {\dot{\lambda }}_{k-1}\\ \varphi_{k-1}+{\dot{\varphi }}_{k-1}T_{k-1}/60\\ {\dot{\varphi }}_{k-1} \end{array}\\ a_{k-1} \end{array}\right]+\left[\begin{array}{ccc} {\left(T_{k-1}\right)}^{2}\sec(\varphi_{k-1})/120 & 0 & 0\\ T_{k-1} & 0 & 0\\ 0 & {\left(T_{k-1}\right)}^{2}/120 & 0\\ 0 & T_{k-1} & 0\\ 0 & 0 & 1 \end{array}\right]\left[\begin{array}{l} {\ddot{\lambda }}_{k-1}\\ {\ddot{\varphi }}_{k-1}\\ 0 \end{array}\right]$$

with $T_{k-1}=t_{k}-t_{k-1}$. The Equation (19) is nonlinear, so the first-order extended Kalman filter (EKF) is used for state prediction and update.

#### 2.3.2. MHT Tracker

After ship detection, we can get a preliminary detection result for each frame, which contains not only the true targets, but also the false detections such as broken clouds, waves, and other interference. Therefore, a stable tracker is needed for effective data association. In this paper, an MHT tracker with amplitude information is used for ship tracking. MHT algorithm which is regarded as theoretically the best data association algorithm is widely used in modern tracking system. The key idea of MHT is to use multi-frame measurements to resolve the uncertainty of data association hypotheses which are propagated into the future to accumulate the temporal information. The log likelihood ratio (LLR) in MHT is often used to evaluate alternative track formation hypotheses and track score for track jth at frame k can be obtained by the recursion given below:

(20) $$L^{ij}(k)=L^{ij}(k-1)+\Delta L^{ij}(k)$$

where $\Delta L^{ij}(k)$ is the increments of the score, which is calculated as

(21) $$\Delta L^{ij}(k)=\left\{ \begin{array}{l} \ln(1-P_{D}),no\;update\\ \Delta L_{K}^{ij}(k)=\ln\left(\frac{P_{D}}{2\pi \lambda_{f}\sqrt{\left|S\right|}}\right)-\frac{d_{ij}{}^{2}}{2},trackupdate \end{array}\right.$$

where $P_{D}$ is the probability of detection, $\lambda_{f}$ is false alarm density, S is residual covariance matrix, $d_{ij}$ is the Mahalanobis distance between measurement i and track j. To increase data association, the amplitude information is incorporated into the traditional MHT framework in this paper. As target amplitude is independent from its kinematics, the joint likelihood of a target is the product of the kinematic likelihood and the amplitude likelihood, and the original LLR increment can be represented as

(22) $$\Delta L^{ij}(k)=\Delta L_{K}^{ij}(k)+\Delta L_{A}^{ij}(k)$$

where $\Delta L_{K}^{ij}(k)$ and $\Delta L_{A}^{ij}(k)$ are respectively the kinematics and amplitude increment, both of which contribute sufficient information to the track score and data association. In this paper, $\Delta L_{A}^{ij}(k)$ takes the form as

(23) $$\Delta L_{A}^{ij}(k)=\ln\{\exp[-{\left(a^{i}-amp^{j}\right)}^{2}/\sigma_{a}^{2}]/c_{1}\}$$

where $a^{i}$ is the amplitude of measurement i, and $amp^{j}$ is the estimated amplitude of track j. $c_{1}$ is the constant probability for the posterior of the background (null) hypothesis.

As some false targets are also contained in the tracking, the gating test with a distance threshold and the limit of target speed (maximum and minimum) is used. We also use well-known M/N logic method for track management. A tentative track is confirmed if there are at least M detections falling in the subsequent gates over N consecutive scans, otherwise the track is discarded. A confirmed track is terminated if no detections in the past L consecutive frames have been validated or the speed of a target is unfeasible.

## 3. Experiments and Results

### 3.1. Dataset

**Figure 3:**
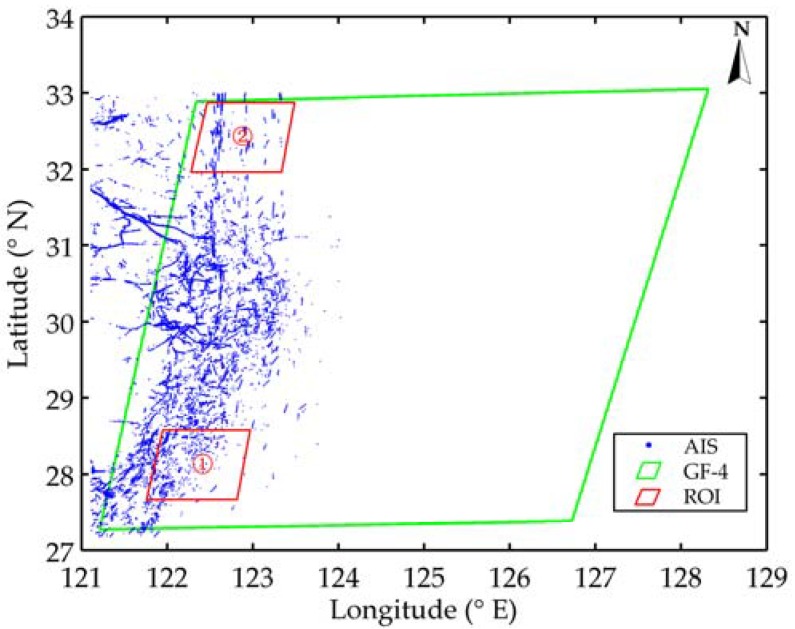
Coverage area of GF-4 satellite image with AIS data. The locations of AIS reports are marked by blue dots. The imaging area is bounded by green lines while two ROIs are bounded by red lines. ROI 1 and ROI 2 are labeled with ① and ②, respectively.

To validate the proposed algorithm, a set of GF-4 sequential images has been used. The imaging area is in the East China Sea as shown in Figure 3, and detailed information of these data sets is given in Table 1. There are 5 frames, with a footprint of approximately 500 km × 500 km. Each data has five spectral bands, and the size of each band is 10,240 pixels × 10,240 pixels with a resolution of 50 m. The level of all images is level 1A, which is not corrected geometrically. As the imaging area is so large and there are hundreds of ships in a single image, it is hard to evaluate our algorithm in the whole region. In this paper, we select two regions of interest (ROI) bounded by red lines to validate the tracking method. The size of each ROI is 2000 pixels × 2000 pixels (about 100 km × 100 km). Meanwhile, AIS data (ground truth) used for position correction is mainly obtained from the shore-based AIS stations. The time range is from 2017-03-09 03:40:00 to 2017-03-09 04:10:00, and the refresh rate of data is up to 1 min.

### 3.2. Evaluation

#### 3.2.1. Quantitative Evaluation Metrics

To validate ship detection and tracking results, the performance of the proposed algorithm is quantitatively evaluated by using the standard evaluation measures: precision, recall and F-score. F-score is the harmonic mean of precision and recall. The precision, recall, and F-score are calculated as

(24) $$precision=\frac{TP}{TP+FP}$$

(25) $$recall=\frac{TP}{TP+FN}$$

(26) $$F-score=\frac{2\times precision\times recall}{precision+recall}$$

where TP, FP and FN are true positives, false positives, and false negatives, respectively.

#### 3.2.2. Quantitative Evaluation

**Figure 4:**
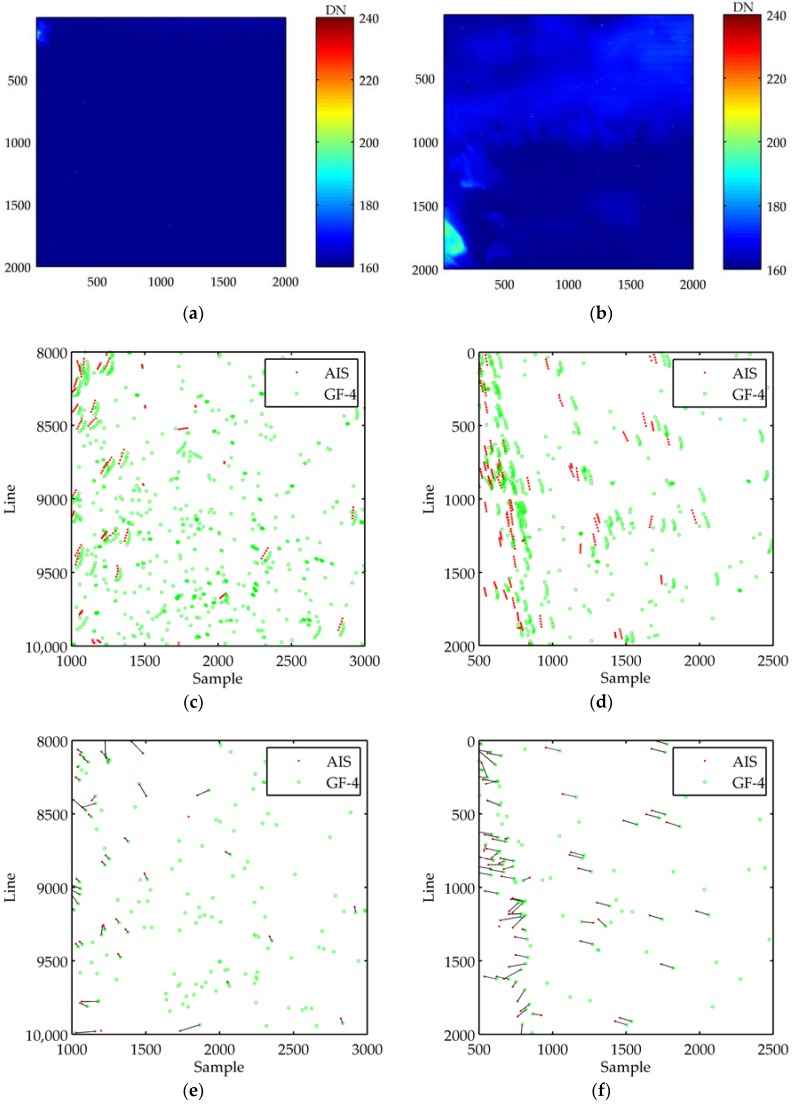

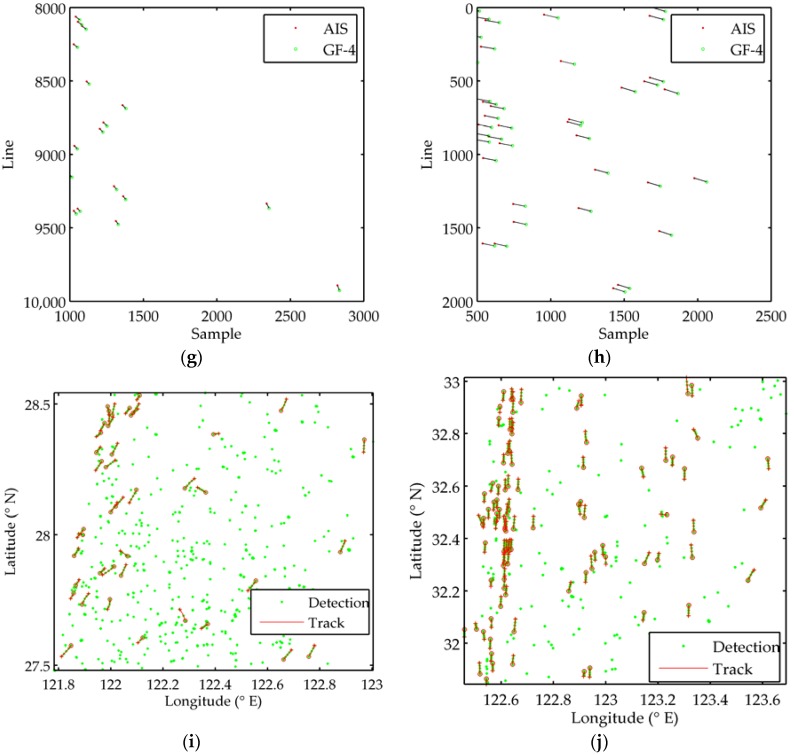
Detection and tracking results in two ROIs. (**a**,**b**) Intensity image in ROI 1 and ROI 2, respectively; (**c**,**d**) Results of ship detection in image coordinates in five frames; (**e**,**f**) GNN data association between AIS and GF-4detection in a single frame; (**g**,**h**) Point pairs after RANSAC processing; (**i**,**j**) Results of ship tracking in geographic coordinates using improved MHT.

Figure 4 is the results of ship detection and tracking in two ROIs. In ship detection, the larger the threshold th of the saliency map is, the smaller the recall is and the larger the precision is. The empirical value of the threshold is 3~5, and it is set to 4 in the experiment. In Figure 4a,b, the units of intensity image are digital numbers (DN). From Figure 4a–d, we can see that the ROI 1 is homogeneous, but a lot of false targets are existed after ship detection. The ROI 2 is an inhomogeneous and traffic-intensive region where there are a lot of ships. In Figure 4c–h, ‘AIS’ and ‘GF-4‘ mean the locations of AIS data and the locations of GF-4 detection reports, respectively. There is absolute positional deviation between the GF-4 detections and the ground truth locations directly using RPCs and the errors are about 10–60 pixels in the sample and line. As shown in Figure 4c,d, we can also see that the points of ship sailing look like curved instead of straight. This is because there is a position deviation between GF-4 satellite sequential images. Therefore, it is necessary to correct the position error before ship tracking. The results of GNN data association in the first frame and matching point pairs after RANSAC processing are shown in Figure 4e–h. The distance threshold ε in GNN association is set to 200, and the number of iteration in RANSAC processing is set to 1000. From the comparison of the subfigures, we can see that the performance of RANSAC is good and it can remove the erroneous point pairs effectively.

In ship tracking, the main parameters are set as: $\sigma_{p}=0.002{}^{\circ}$, $\sigma_{v}=0.01$ nm/h^2^, $P_{D}=0.95$, $\lambda_{f}=10^{-11}$, $\sigma_{a}=15$, $c_{1}=0.1$. For track management, the 3-of-4 test is used to confirm tracks and tracks are terminated after 2 missed detections. Figure 4i,j shows the tracking results in geographical coordinates after RPCs correction, where ‘Detection’ and ‘Track’ mean the detection reports (measurements) and the tracking results of our algorithm, respectively. A red line represents a track, where ‘o’ and ‘+’ represent the start and of end of a track, respectively.

Table 2 reports the calculated metrics of detection and tracking in five frames in two ROIs. TP, FP and FN are confirmed by manual identification and AIS information comparison analysis. There are total 151 ships (53 ships in ROI 1 and 98 ships in ROI 2) in two ROI, and 79 tracked ships (22 ships in ROI 1 and 59 ships in ROI 2) is verified by real-time AIS data. From Table 2, we can see that there are many false targets before ship tracking. The false alarm rate is high in region 1 mainly because some inferences such as ocean waves are similar to ships. It is satisfactory that the total recall in two ROIs is above 80%. The false negatives (missing targets) in ship detection are small-size ships whose lengths are 50~100 m by the analysis of unassociated AIS data. Through MHT tracking algorithm, false targets can be reduced effectively, and true positives can be estimated accurately. The total F-score after tracking are both above 90%. Moreover, the improved MHT which combines target amplitude can improve measurement-to-track data association. The improved tracker reacts less sensitive to false positives than the MHT only using position and has higher precision.

**Figure 5:**
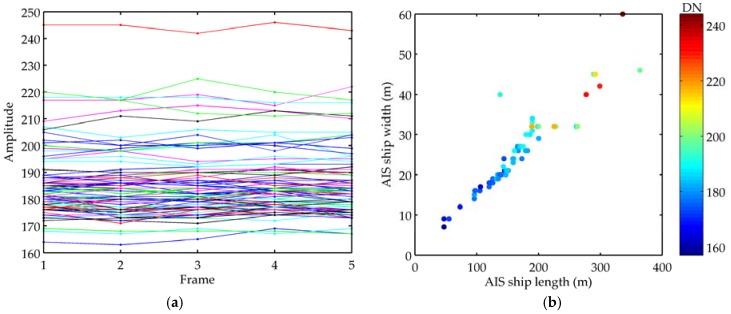
Amplitude information of ships. (**a**) Amplitudes of tracked ships in five frames. Different colored lines represent different ships; (**b**) Mean amplitudes of tracked ships compared with the sizes of ships verified by AIS data.

Figure 5 is the amplitude information of tracked ships. In Figure 5a, the amplitudes of the same target in five frames are represented by the same color. It can be seen from that the ship’s amplitude is stable and distinguished. This is also the reason we use amplitude information for improving tracking performance. Figure 5b is the mean amplitudes of tracked ships compared with ship sizes in AIS data. We can see that the larger the size of the ship, the greater the amplitude value, and easier to be detected. The range of lengths of ships is from 40 m to 400 m, and even some ships whose lengths are below 50 m (less than the image spatial resolution of the GF-4 satellite) can be identified after ship detection and tracking. From another perspective, it also reflects the strong capabilities of the GF-4 satellite for marine surveillance.

**Figure 6:**
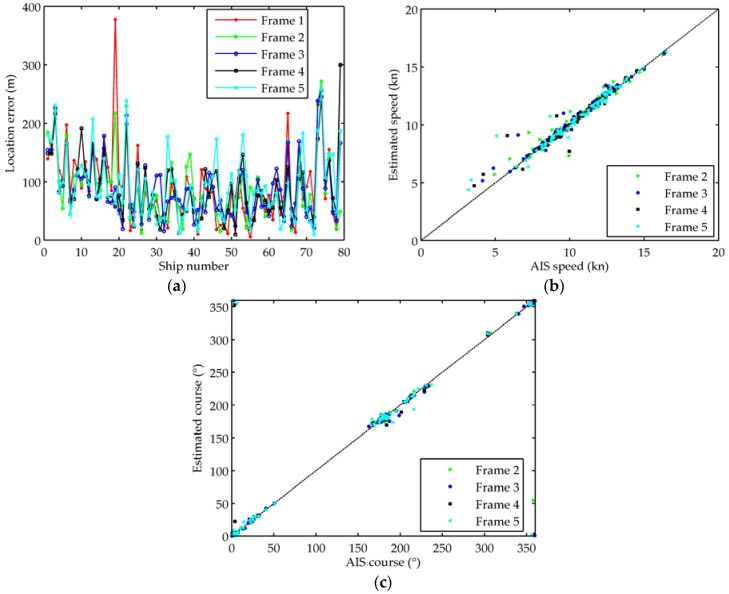
Comparisons of the motion estimation with AIS data. (**a**) Absolute error between estimated location and AIS location; (**b**) Estimated speed compared with AIS speed; (**c**) Estimated course compared with AIS course.

79 ships verified by AIS data in two ROIs are used to assess the motion state. Figure 6 shows the comparisons of motion state estimation between the tracking results and AIS data in five frames. The average absolute error of motion estimation about ships is reported in Table 3 in detail. The tracking accuracy is accurate and improved largely after position correction with AIS data. The total location error is reduced from 3800 m to 90 m. The speed and the course are well estimated by our method, and the average error of speed and course are 0.3 kn (approximately 0.15 m/s) and 2.5°, respectively. Meanwhile, it can also be seen from Figure 6b that the estimated accuracy of speed is improved with the increase of speed. The accuracy of motion estimation can basically meet the needs of marine surveillance, and illustrates the high-efficiency of the proposed method. The results presented in the experiment are obtained with a laptop (Intel Core i5, CPU 2.5 GHz, RAM 6 G) using MATLAB 2010a software. In each ROI, the average processing time from ship detection to ship tracking is approximately 50 s per frame, which shows that our algorithm has the potential for near real-time processing.

## 4. Conclusions

The GEO remote sensing satellite has wide application prospects in sea surveillance over a wide area, and the GF-4 satellite has the highest spatial resolution in GEO satellites. In this paper, a ship-tracking algorithm applied to the GF-4 satellite is proposed, which is designed based on ship detection, position correction and ship-tracking three-step processing. A simple saliency method based on PSNR is designed for ship detection. Ship positioning accuracy is improved by RPCs model using AIS data as dynamic GCPs. Finally, ship tracking and motion parameter estimation in geographic coordinate are realized using the MHT tracker aided by amplitude information. The tracking performance is analyzed using GF-4 sequential images by calculation of recall, precision and so on. The experiment results show that the proposed algorithm has better performance with high detection rates, low false alarm rate, high association rate and high tracking precision. However, there are still potential developments to improve the accuracy of detection and tracking, especially for small ships. Using information (such as ships’ spectral and wake information), tracking before detection (TBD) and machine learning methods will be pursued in our future work.

The proposed algorithm also has potential applications in other research areas, such as target tracking in small satellite videos. As a single sensor cannot solve all problems in space-borne maritime surveillance, multi-sensor information fusion based on GEO medium resolution remote sensing satellites, LEO high-resolution remote sensing satellites, AIS satellites and signals intelligence (SIGINT) satellites, electronic intelligence (ELINT) satellites and other assets is the trend of future development. It is also challenging work worth studying.

## Figures and Tables

**Table 1:** Details of the GF-4 data sets.

Frame	Acquisition Time (UTC)	Weather and Sea Conditions	
		Wind Scale	Sea State Scale
1	2017-03-09 03:47:24	3~4	2~3
2	2017-03-09 03:50:30		
3	2017-03-09 03:53:36		
4	2017-03-09 03:56:42		
5	2017-03-09 03:59:47		

**Table 2:** Ship detection and tracking performance of the experiment.

ROI	Before Tracking			After Tracking (Only Position)			After Tracking (Position + Amplitude)		
	Precision	Recall	F-Score	Precision	Recall	F-Score	Precision	Recall	F-Score
1	26.6%	79.6%	39.9%	93.0%	75.5%	83.3%	97.6%	77.3%	86.3%
2	74.5%	94.1%	83.2%	97.8%	91.8%	94.7%	99.0%	92.9%	95.9%
Total	47.6%	89.0%	62.0%	96.3%	86.1%	90.9%	98.5%	87.4%	92.6%

**Table 3:** Average error of motion estimation.

ROI	Before Position Correction			After Position Correction		
	Location Error (m)	Speed Error (kn)	Course Error (°)	Location Error (m)	Speed Error (kn)	Course Error (°)
1	1804.7	0.7	6.3	123.9	0.2	3.0
2	4613.9	1.1	3.7	77.4	0.3	2.3
Total	3816.7	1.0	4.4	89.8	0.3	2.5
